# A Hybrid Automata Approach for Monitoring the Patient in the Loop in Artificial Pancreas Systems

**DOI:** 10.3390/s21217117

**Published:** 2021-10-27

**Authors:** Aleix Beneyto, Vicenç Puig, B. Wayne Bequette, Josep Vehi

**Affiliations:** 1 Department of Electrical, Electronic and Automatic Engineering, University of Girona, 17004 Girona, Spain; aleix.beneyto@udg.edu; 2 Automatic Control Department-Campus de Terrassa, Universitat Politècnica de Catalunya (UPC), 08222 Terrassa, Spain; vicenc.puig@upc.edu; 3 Department of Chemical Engineering, Rensselaer Polytechnic Institute, Troy, NY 12180, USA; bequette@rpi.edu; 4 Centro de Investigación Biomédica en Red de Diabetes y Enfermedades Metabólicas Asociadas, 28029 Madrid, Spain

**Keywords:** artificial pancreas, hybrid automaton, Kalman filter, patient in the loop, type 1 diabetes

## Abstract

The use of automated insulin delivery systems has become a reality for people with type 1 diabetes (T1D), with several hybrid systems already on the market. One of the particularities of this technology is that the patient is in the loop. People with T1D are the plant to control and also a plant operator, because they may have to provide information to the control loop. The most immediate information provided by patients that affects performance and safety are the announcement of meals and exercise. Therefore, to ensure safety and performance, the human factor impact needs to be addressed by designing fault monitoring strategies. In this paper, a monitoring system is developed to diagnose potential patient modes and faults. The monitoring system is based on the residual generation of a bank of observers. To that aim, a linear parameter varying (LPV) polytopic representation of the system is adopted and a bank of Kalman filters is designed using linear matrix inequalities (LMI). The system uncertainty is propagated using a zonotopic-set representation, which allows determining confidence bounds for each of the observer outputs and residuals. For the detection of modes, a hybrid automaton model is generated and diagnosis is performed by interpreting the events and transitions within the automaton. The developed system is tested in simulation, showing the potential benefits of using the proposed approach for artificial pancreas systems.

## 1. Introduction

Type 1 diabetes (T1D) is a serious metabolic disease characterized by an autoimmune destruction of the insulin-producing β-cells in the pancreas and subsequent insulin deficiency. Insulin is a hormone that allows glucose uptake from the blood into cells, either to be used as fuel or stored for future use. Low levels of insulin inevitably lead to high blood glucose (BG) concentrations, known as hyperglycemia, which can also lead to long-term complications [1,2]. Current therapies are based on administering exogenous insulin using devices such as insulin pumps or pens [3,4].

Artificial pancreas (AP) is a closed-loop (CL) system in which insulin is delivered automatically by adjusting a pump’s insulin infusion rate depending on continuous glucose monitor (CGM) readings [5]. CGM sensors measure glucose subcutaneously and provide an estimate of current BG levels. Many different control strategies for AP are available in the literature [5,6,7]. AP systems have been an important focus of research and discussion over the last years, with a multitude of clinical trials being conducted world-wide [8]. There is clinical evidence that suggests that using an AP is safe, robust, and efficacious for people with T1D when compared to traditional open-loop therapy [7,8].

However, AP technology is not exempt of risks during operation [9]. It is especially critical to take into account the concept of patient-in-the-loop in the design phase for this kind of technology [10], because ultimately, patients will wear and operate APs. Patients will have to provide inputs, maintain the system, and ideally undertake training before using any AP. Therefore, the patient will play a significant role in any configuration—a clear example is the Medtronic MiniMed 670G AP [11]. Undoubtedly, research is trying to find solutions that reduce the impact of patient behaviors and decisions. Therefore, any developed AP must implement fault tolerant control (FTC) strategies to mitigate faults and ensure and maintain stability, performance, and safety for the patient.

In the context of FTC research for AP systems, most of the literature is focused on fault detection (FD) and fault identification (FI) for either the CGM [12,13] or insulin pumps [14,15,16,17]. Only few tackle the issue of how the patient-in-the-loop affects the system and what potential faults may arise from it [9]. Developed strategies to detect specific patient behaviors exist, however, they were not developed for safety but as ad-hoc approaches for control. Most of the approaches involving the patient estimate the glucose rate of appearance to allow AP to operate without meal announcement [18,19,20,21,22,23]. Other strategies try to detect when patients exercise and modify or adapt their control strategy accordingly [24,25]. In any case, the roads being followed are either hybrid systems, which rely on patient input, or fully CL systems. The patient-in-the-loop paradigm links both approaches allowing fully CL systems to operate, but also to rely on contrasted patient input information to enhance performance and safety. Hence, patient modes and inputs should be monitored and potential faults must be uncovered for fully CL systems that may from time to time may use patient information. Here, a patient operational mode is defined by its significantly different system dynamics, for example the increased insulin sensitivity during aerobic exercise. Additionally, patient inputs are considered as any action that the patient can perform with direct implications to BG control.

The aim of this work is to provide robust and safe solutions to deal with the patient-in-the-loop in AP systems. In the context of diabetes and AP systems, the patient should be considered as part of the control loop [26]. Depending on the system configuration, the patient may have to take the role of an actuator or a sensor, besides being the plant to control. Specifically, the subjects will be considered actuators performing the control action of eating the recommended carbohydrates (CHO) [27] or informing about meal consumption or exercise. It is known that patient information can be erroneous, for example, studies show that patients have significant estimation errors when counting CHO [28]. Therefore, the detection and monitoring of patient modes can be used to help the control system in maintaining performance and safety despite patient faults. Considering the patient-in-the-loop, the monitoring of system modes can be aided by the feed-forward information provided by the patient. That information is inherently affected by uncertainty and must be checked to exclude any possible fault.

The implemented approach is a model-based FD system and can work with any kind of hybrid or fully CL control architecture that announces meals and/or uses insulin and/or CHO as a control action. The physiology of a person with T1D is subjected to highly nonlinear phenomena. A way of extending traditional linear system theory to nonlinear systems is by means of the linear varying parameter (LPV) paradigm [29]. Here, this approach will be used for modeling T1D subjects. Then, we design a bank of polytopic Kalman filters by solving the dual of the linear quadratic control (LQC) problem and imposing constraints on the system stability and performance by using linear matrix inequalities (LMI). Dealing with system uncertainties in model-based approaches is known to be an important factor. In this work, we use a deterministic approach using zonotopes to build interval observers (IO). The resulting bank of IO will be used to generate a set of residual signals online. A hybrid automaton (HA) is built based on a configuration of normal and faulty patient modes. Transitions between modes are done by analyzing the patient input events and checking the residuals consistency.

This paper is organized as follows: Section 2 presents the problem formulation and briefly describes the control system. Section 3 describes the patient monitoring system. Section 4 describes the modeling process of a T1D patient. Section 5 is focused on the residual generation by designing IO. In Section 6, in-silico benchmarks are proposed to evaluate the monitoring system. Finally, Section 7 and Section 8 include the discussion and conclusions from this work.

## 2. Problem Statement

The Spanish consortium on artificial pancreas and diabetes technology (eSCAPE) has developed a multivariable hybrid AP system for the regulation of glucose to cope with the two disturbances that have the biggest effect on BG, meals, and exercise. The system uses both insulin and CHO as control actions to maintain BG in the euglycemic range 70–140 mg/dL. The insulin feedback loop is based on a proportional-derivative (PD) controller with insulin feedback (IFB) that integrates a safety layer with insulin-on-board (IOB) constraints and sliding mode reference conditioning (SMRC) [30,31]. The second feedback loop uses a predictive PD controller with a quantization system that encourages the patient to consume CHO if there is danger of hypoglycemia [27]. Both control loops are coordinated by using coupled carbohydrates-on-board (COB) inhibition signals that ensure that insulin and CHO control actions are effective and not mutually counteracted.

**Figure 1:**
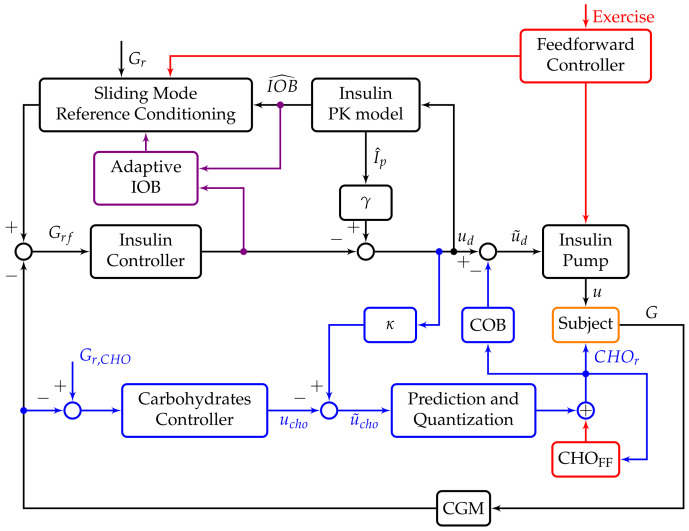
Multivariable hybrid AP. The black blocks and signals are elements from the insulin feedback loop, the blue from the CHO feedback loop, the red from the feed-forward exercise control, the violet is the IOB adaptive algorithm and the orange block is the patient to control.

The system has recently been upgraded with additional modules to enhance performance and safety when exercise is announced. If patients announce exercise, a feed-forward controller will adapt the insulin gains and, if required, the consumption of a snack will be suggested [32]. Furthermore, we included an adaptive IOB system to enhance postprandial control [33]. This control system has already been tested in clinical trials against meals and exercise and has showed promising results [34,35,36]. The overall control strategy is depicted in Figure 1 and is the control system that will be used in this work.

This system is a hybrid AP where the patient takes fundamental roles. We want to address the patient-in-the-loop situation that arises when using patients as an operator and as the plant to control. In this configuration, patients play a sensor role when announcing meals and exercise. They also play an actuator role when the CHO controller suggests rescue CHO. Therefore, patients can introduce errors into the system that could eventually lead to faults. We consider patient-in-the-loop faults as poorly estimated meal boluses, when patients do not follow the CHO controller recommendations, or when the dynamical plant is behaving in an unexpected manner.

## 3. Materials and Methods

In this work, we develop a system for the detection of patient modes by using the scheme proposed in [37] based on creating a HA model that presents as many states as patient modes. For mode detection, we will generate a set of residual signals. Checking at every time instant which residuals are consistent with the current mode, we can detect the change of mode. Residuals will be generated by using a bank of zonotopic Kalman filters.

### 3.1. Hybrid Automaton

**Figure 2:**
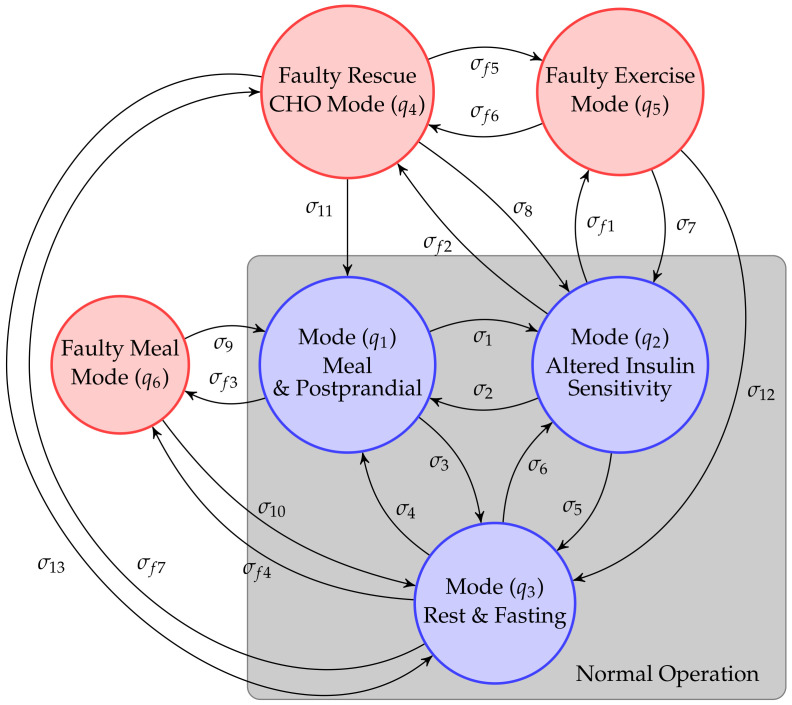
Component automata. The hybrid model of the automata includes three normal operation modes and considers three faulty modes.

The HA has been developed based on [37]. The idea behind using an HA is to mimic real patient operational modes and transitions. Using this approach, the HA is defined by the following components, $HA^{k}=<Q,X,U,Y,F,G,H,\Sigma ,T>$ where:

1. Q is the set of modes. Each of the modes $q_{i}\in Q$ represents an operational mode of the patient. The set of modes is constituted by nominal and faulty operational modes, i.e., $Q=Q_{N}\cup Q_{F}$. Particularly, we consider three nominal operational modes and three faulty modes, see Figure 2;
2. $X\subseteq R^{n_{x}}$ defines the state space of the system on each of the modes x(k)∈X, where x(k) is the state space vector. $U\subseteq R^{n_{u}}$ and $Y\subseteq R^{n_{y}}$ define the continuous input and output spaces. In this work, the input space includes both insulin, rescue CHO and meal inputs, and the output space only considers the CGM measurements. G defines the set of discrete time state functions and H is the set of discrete time output functions;
3. $\Sigma =\Sigma_{S}\cup \Sigma_{C}\cup \Sigma_{F}$ is a set of events. This set of events can be split into spontaneous mode switching events $\Sigma_{S}$, input events $\Sigma_{C}$ and fault events $\Sigma_{F}$. Spontaneous switching events are unknown events that may produce transitions between modes in the real patient. For example, an unannounced meal may lead to a spontaneous transition to the meal operational mode, even if that event (eating) is unknown to the system. Input events are defined by patient actions, such as announcing meals or exercise. Fault events are defined by checking the consistency of the residuals and the ending transitioning mode;
4. F is the set of possible faults. For each faulty mode $q_{i}\in Q_{F}$ the system has a specific $f_{i}\in F$, which is associated with a fault event $\Sigma_{F}$;
5. T:Q×Σ→Q is the transition function. Transitions from one mode $q_{i}$ to another mode $q_{j}$ are labeled by an event σ∈Σ. Transitions labeled as $\sigma_{f}\in \Sigma_{F}$ indicate that the transition is a faulty transition.

The HA operational modes and transitions for the patient are presented in Figure 2. Three automata modes define the normal operation for the system. These modes were selected for its differentiated dynamics and significance during operation. Transitions between and to these modes are labeled $\Sigma_{S}\cup \Sigma_{C}=\left\{\sigma_{1},\sigma_{2},\cdots ,\sigma_{13}\right\}$ and include input patient information about meals and exercise, and events triggered by the consistency of residuals. Transitions to faulty modes are possible depending on the current operation mode. The set $\Sigma_{F}=\left\{\sigma_{f1},\sigma_{f2},\cdots ,\sigma_{f7}\right\}$ represent the fault events. Some of these faults may be structural (faults in the actuator) such as not eating rescue CHO or injecting inappropriate boluses for meals.

### 3.2. Online Diagnoser

The online diagnoser is responsible of the mode change detection within the HA. At every sampling time *k*, the patient observable events $\Sigma_{C}$ and the generated residuals are processed and used by the hybrid diagnosis to detect specific modes or faults. Patient-in-the-loop faults are detected when a triggered transition leads to one of the faulty modes, see Figure 2.

The consistency of the residuals is checked at every sampling time against the designed thresholds. A potential mode change is detected if the residuals violate any of the thresholds. Then, a binary signature vector (F) is generated and checked with the admissible rows of the fault signature matrix (FSM). The FSM is a binary matrix that displays the signatures required to move from one automaton mode to another. A signature is used to describe a configuration of symptoms that may lead to a transition. Each of the rows of the FSM are related to each of the possible automaton transitions T. Hence, the FSM has as many rows as possible events σ ($n_{\sigma }=20$) and as many columns as existing automaton modes ($n_{m}=6$).

(1) $$\mathrm{FSM}=\left[\begin{array}{cccc} f_{1,1} & f_{1,2} & \cdots & f_{1,n_{m}}\\ f_{2,1} & f_{2,2} & \cdots & f_{2,n_{m}}\\ \vdots & \vdots & \vdots & \vdots \\ f_{n_{\sigma },1} & f_{n_{\sigma },2} & \cdots & f_{n_{\sigma },n_{m}} \end{array}\right]$$

This can be viewed as having a total of $n_{m}$ different subsets of FSMs for each of the HA modes. For example, the FSM associated to the mode $q_{1}$ is as follows

(2) $${\mathrm{FSM}}_{q_{1}}:=\left[\begin{array}{cccccc} 0 & f_{\sigma_{1},q_{2}} & 0 & 0 & 0 & 0\\ 0 & 0 & f_{\sigma_{3},q_{3}} & 0 & 0 & 0\\ 0 & 0 & 0 & 0 & 0 & f_{\sigma_{f3},q_{6}} \end{array}\right]=\left[\begin{array}{cccccc} 0 & 1 & 0 & 0 & 0 & 0\\ 0 & 0 & 1 & 0 & 0 & 0\\ 0 & 0 & 0 & 0 & 0 & 1 \end{array}\right]$$

The **FSM_q1_** will only be used when the current HA mode is $q_{1}$. Then, the binary signature vector $F_{1\times n_{m}}$ is constructed at every sampling time. Elements $f_{i}$ of F are computed as

(3) $$f_{i}=\left\{\begin{array}{cc} 1, & if\prod_{i=1}^{M}\left(t_{r_{i}}^{l}<0\vee t_{r_{i}}^{u}>0\right)=1\\ 0, & otherwise \end{array}\right.$$

where $i=1,\cdots ,n_{m}$ indicates the binary residual generated by the appropriate observers for the current operation mode at time instant *k*, $M\leq n_{o}$ accounts for possible residual combinations that may be used to generate the binary signal, $n_{o}$ is the total number of observers, $t_{r_{i}}^{l}$ and $t_{r_{i}}^{u}$ are the lower and upper interval residual bands generated by the *i*-th zonotopic observer. Essentially, if the observer predicted band does not include the zero residual, then the real system behavior cannot be explained and a fault is triggered by that residual signal. The vector **F** is then compared element-wise to each of the rows of the appropriate **FSM_$q_{i}$_**. If the signature **F** is not matched by any of the rows of the particular **FSM_$q_{i}$_**, then transition detection is not possible and the HA stays at the current mode.

### 3.3. Observable Events Processing

Using a binary transition and fault detection system might not be enough for detection if several signatures have the same binary combination, i.e., they are not isolable. For that reason, the transitions may be aided with external information, such as input events, the sign of the residuals and by the value and trend of the measured variables. In this work, the system makes use of patient information, if available, such as meal/exercise announcements. Particularly, we consider the following transitions based on the FSM and patient information:

1. *Meal and Postprandial Mode Detection*: Transitions between the meal/postprandial mode ($q_{1}$) and the resting/fasting mode ($q_{3}$) happen when in normal operation. If meals are announced, the system can instantly switch to the meal/postprandial mode and if in a designed period of 15 min the meal observer does not explain the system behavior, a transition to the meal faulty mode ($q_{6}$) will be triggered. In the situation where both meal and rest residuals are consistent, the system returns to the resting/fasting operational mode.
2. *Altered Sensitivity Periods Detection*: Transitions $\sigma_{1}$, $\sigma_{2}$, $\sigma_{5}$, and $\sigma_{6}$ to and from the changed insulin sensitivity mode ($q_{2}$) are considered. Similarly to $\sigma_{4}$, the transition events $\sigma_{1}$ and $\sigma_{6}$ are activated if the altered sensitivity residual is consistent with the observations. In this approach, an altered sensitivity period is caused when performing aerobic physical activity. Then, if exercise is announced, a transition to the $q_{2}$ mode can instantly happen if the altered sensitivity residuals are consistent. Transition events $\sigma_{2}$ and $\sigma_{5}$ are triggered if the meal or the rest residuals consistency are valid and the altered insulin sensitivity residual is not. If the consistency of both residuals generated by the meal and rest observers are satisfied, then the binary FSM cannot tell which transition to execute. In such a case, prior patient inputs can be used to decide if a transition needs to be made. If the residual from the altered sensitivity observer is no longer consistent, the residual with minimum absolute value is picked as the next transition event to be processed.
3. *Misestimated Meals Detection*: The AP used in this work requires patients to announce meals. Announcing a meal means that the patient needs to provide two inputs: (1) the time when the meal is going to be consumed and (2) the quantity of CHO in grams of that meal. The detection of a misestimated meal ($q_{6}$) involves the transition events $\sigma_{9}$, $\sigma_{10}$, $\sigma_{f3}$ and $\sigma_{f4}$. Transition towards $q_{6}$ is possible through events $\sigma_{f3}$ and $\sigma_{f4}$. If a meal is not announced while being on mode $q_{3}$ and the rest observer is not consistent, then the CGM signal is checked. If CGM is higher than 160 mg/dL and the trend for the last 30 min is positive, a transition to mode $q_{6}$ happens. Contrarily, if the meal is announced, then transition event $\sigma_{f3}$ might be triggered when in mode $q_{1}$, if the meal observer is not consistent.
4. *Missed Rescue Carbohydrates Detection*: Two types of rescue CHO are considered: (1) feed-forward rescue CHO when exercise is announced and (2) rescue CHO suggested by the feedback controller. This control action is a source of potential patient-in-the-loop faults in free living conditions. Patients may forget to consume the suggested CHO or they will simply not consume them for other physiological reasons, such as weight gain. Transitions to the faulty rescue CHO mode ($q_{4}$) are expected through events $\sigma_{f6}$ or $\sigma_{f7}$, which are only checked when the controller triggers a recommendation of CHO. Transitions $\sigma_{8}$, $\sigma_{11}$ and $\sigma_{13}$ return the HA to normal operational modes, respectively to $q_{2}$, $q_{1}$ and $q_{3}$. These transitions are exclusive, meaning that if any of the meal, rest or altered sensitivity observer residuals are consistent, a transition to any of the aforementioned normal operation modes will be triggered.

## 4. Modeling of T1D Patients

In this section, the model used for the design of observers is presented. Several dynamical models are available in the literature to describe normal and impaired glucose metabolism in humans. One of the early models and most likely the most used is the so-called minimal model [38], which only consists of three state variables and is able to capture some of the underlying glucose dynamics. Since then, many other models have appeared such as the more complex Food and Drug Administration (FDA) accepted UVA/Padova T1D Simulator [39]. Selecting a model for the design of observers is crucial and one has to trade off precision and complexity with simplicity and usability. Particularly, complex glucose models may present observability issues because only one variable is measured. For that reason, we use a modified version of the Hovorka nonlinear model [40], which is of intermediate complexity [41].

### 4.1. The Reduced Hovorka Model

**Figure 3:**
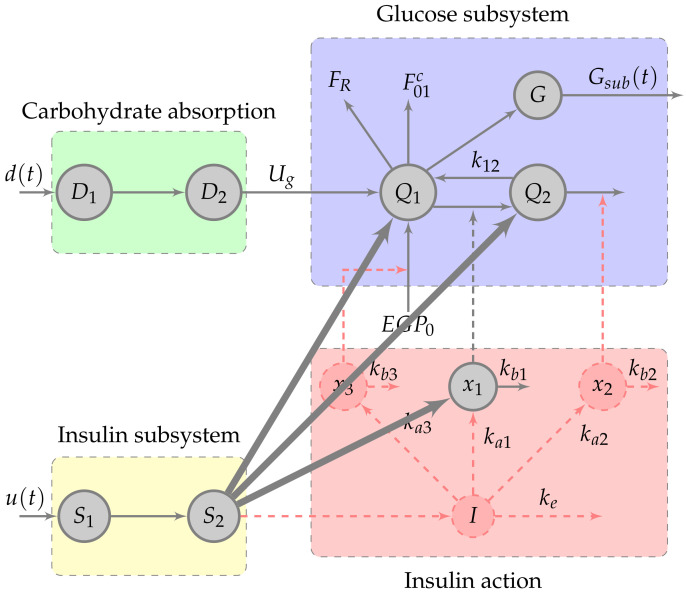
Block diagram of the reduced Hovorka nonlinear model. The dashed red nodes and arrows indicate the structural relations removed from the original nonlinear Hovorka model. The thicker black lines denote new relations in the reduced model.

The model used in this work is a reduced version of the nonlinear Hovorka model [40] as proposed in [42]. Figure 3 shows the whole block diagram of both model versions. It is a compartmental model with four main sub-systems: carbohydrate absorption, subcutaneous insulin absorption, insulin action on glucose uptake and removal, and the BG dynamics.

The simplified model excludes the states I(t), $x_{2}\left(t\right)$ and $x_{3}\left(t\right)$. By doing this reduction, not only have we decreased the system complexity, but most importantly we have improved the structural behavior of the overall model. Notice the new direct connections from node $S_{2}$ to $Q_{1}$ and $Q_{2}$. Those connections greatly simplify any observability issues since more relations between different states are considered. The removed states define insulin dynamics and are described by

(4) $$\begin{array}{cc} \dot{I}\left(t\right)= & \frac{S_{2}\left(t\right)}{t_{maxI}V_{i}}-k_{e}I\left(t\right)\\ {\dot{x}}_{2}\left(t\right)= & -k_{a2}x_{2}\left(t\right)+S_{ID}^{f}k_{a2}I\left(t\right)\\ {\dot{x}}_{3}\left(t\right)= & -k_{a3}x_{3}\left(t\right)+S_{IE}^{f}k_{a3}I\left(t\right) \end{array}$$

The reduced model is obtained by considering the steady state value of the removed states into the rest of the equations. One can easily obtain the following steady state relations

(5) $$I^{ss}=\frac{S_{2}}{t_{maxI}V_{i}k_{e}}$$

which leads to

(6) $$\begin{array}{cc} x_{2}^{ss}= & \frac{S_{ID}^{f}}{t_{maxI}V_{i}k_{e}}S_{2}\\ x_{3}^{ss}= & \frac{S_{IE}^{f}}{t_{maxI}V_{i}k_{e}}S_{2} \end{array}$$

Plugging these equations into the original Hovorka model and removing the aforementioned states leads to the final model

(7) $$\begin{array}{cc} {\dot{S}}_{1}\left(t\right)= & u\left(t\right)-\frac{S_{1}\left(t\right)}{t_{maxI}}\\ {\dot{S}}_{2}\left(t\right)= & \frac{S_{1}\left(t\right)-S_{2}\left(t\right)}{t_{maxI}}\\ {\dot{x}}_{1}\left(t\right)= & k_{a1}\left(\frac{k_{a}S_{IT}^{f}}{V_{I}k_{e}}S_{2}\left(t\right)-x_{1}\left(t\right)\right)\\ {\dot{Q}}_{1}\left(t\right)= & -x_{1}\left(t\right)Q_{1}\left(t\right)+k_{12}Q_{2}\left(t\right)-F_{01}^{c}-F_{R}-U_{g}+\\ \; & +EGP_{0}\left(1-\frac{k_{a}S_{IE}^{f}}{V_{I}k_{e}}S_{2}\left(t\right)\right)\\ {\dot{Q}}_{2}\left(t\right)= & x_{1}\left(t\right)Q_{1}\left(t\right)-k_{12}Q_{2}\left(t\right)+\frac{k_{a}S_{ID}^{f}}{V_{I}k_{e}}S_{2}\left(t\right)Q_{2}\left(t\right) \end{array}$$

where u(t) is the exogenous insulin infusion (basal and bolus), $Q_{1}$ and $Q_{2}$ are the masses of glucose in the accessible and non-accessible (not measurable) compartments, respectively, $S_{1}$ and $S_{2}$ define the insulin absorption rate dynamics and $F_{R}$ and $F_{01}^{c}$ are defined as

(8) $$F_{01}^{c}\left(t\right)=\left\{\begin{array}{cc} F_{01} & ifG\left(t\right)\geq 4.5\mathrm{mmol}L^{-1}\\ F_{01}G/4.5 & \mathrm{otherwise} \end{array}\right.$$

(9) $$F_{R}\left(t\right)=\left\{\begin{array}{cc} 0.003\left(G\left(t\right)-9\right)V_{g} & ifG\left(t\right)\geq 9\mathrm{mmol}L^{-1}\\ 0 & \mathrm{otherwise} \end{array}\right.$$

The original Hovorka model assumes that the compartment *G* represents the measurable glucose concentration and defines it as $G\left(t\right)=Q_{1}\left(t\right)/V_{g}$. However, one can use any other CGM model available in the literature.

The glucose absorption sub-system can be defined separately as

(10) $$\begin{array}{cc} {\dot{D}}_{1}\left(t\right)= & -\frac{D_{1}\left(t\right)}{t_{maxG}}+d\left(t\right)B\\ {\dot{D}}_{2}\left(t\right)= & \frac{D_{1}\left(t\right)}{t_{maxG}}-\frac{D_{2}\left(t\right)}{t_{maxG}}\\ U_{g}\left(t\right)= & \frac{D_{2}\left(t\right)}{t_{maxG}} \end{array}$$

where $U_{g}$ is the rate of carbohydrate absorption, d(t) is the input meal content in grams and $D_{1}$ and $D_{2}$ are glucose compartments. If meals are considered in the model, the state vector is simply extended with states $D_{1}$ and $D_{2}$.

### 4.2. Model Parameters and Individualization

For the purpose of state estimation, we use the model constants and mean parameter values presented in [40] and summarized in Table 1.

The model is firstly individualized by the patient’s weight. The patient weight alters the insulin sensitivity parameters and the available glucose and insulin distribution volumes. However, this might not be sufficient to characterize highly varying patients and therefore model predictions will be inherently erroneous. To address this individualization issue, the insulin sensitivity parameters are adapted based on the fasting patient glucose and basal infusion rate. Then, a new tuning parameter η is introduced and obtained by solving the steady state of the original model

(11) $$f(S_{1},S_{2},x_{1},x_{2},x_{3},I,Q_{2},\eta )=0$$

were $f(S_{1},S_{2},x_{1},x_{2},x_{3},I,Q_{2},\eta )$ represents the non-linear model equations and η is a factor that multiplies the insulin sensitivity $S_{IT}^{f}$, $S_{ID}^{f}$ and $S_{IE}^{f}$ terms.

### 4.3. Linear Parameter Varying Model

LPV systems are linear time varying systems whose state-space system matrices depend on a vector of varying parameters. These parameters can be estimated online or measured. Then, the continuous system representation is given by

(12) $$\begin{array}{cc} \dot{x}\left(t\right)= & A(\Phi (t))x(t)+B(\Phi (t))u(t)\\ y(k)= & C(\Phi (t))x(t)+D(\Phi (t))u(t) \end{array}$$

where $A\left(\Phi \left(t\right)\right)\in R^{n_{x}\times n_{x}}$, $B\left(\Phi \left(t\right)\right)\in R^{n_{x}\times n_{u}}$, $C\left(\Phi \left(t\right)\right)\in R^{n_{y}\times n_{x}}$ and $D\left(\Phi \left(t\right)\right)\in R^{n_{y}\times n_{u}}$ are the system state space matrices that depend on the varying time-dependent vector $\Phi \left(t\right)\in R^{l}$. The vector of varying parameters Φ(t) depends at the same time on some measurable signals $\rho \in R^{n_{\Phi }}$, referred to as scheduling variables, that can be estimated using an appropriate scheduling function

(13) Φ(t)=p(ρ(t))

where $p:R^{l}\to R^{n_{\Phi }}$ is a continuous mapping. If the vector of scheduling variables depend on some internal variables, such as internal states, the system is called quasi-LPV [43]. In this work, the system is strictly proper, with input and output matrices being invariant B(Φ(t))=B, C(Φ(t))=C and D(Φ(t))=0.

Particularly, the reduced Hovorka nonlinear model can be transformed into the LPV representation (12) using the nonlinear embedding approach proposed in [43]. This approach is based on embedding the system nonlinearities inside the scheduling parameters of the LPV model. Then, the following state space matrices are obtained for the reduced Hovorka model in LPV form

(14) $$\begin{array}{cc} A(\Phi (t))= & \left[\begin{array}{ccccccc} -k_{a} & 0 & 0 & 0 & 0 & 0 & 0\\ k_{a} & -k_{a} & 0 & 0 & 0 & 0 & 0\\ 0 & a_{31} & -k_{a1} & 0 & 0 & 0 & 0\\ 0 & a_{41} & a_{43} & a_{44} & k_{12}+a_{45} & 0 & a_{47}\\ 0 & a_{52} & a_{53} & 0 & -k_{12} & 0 & 0\\ 0 & 0 & 0 & 0 & 0 & a_{66} & 0\\ 0 & 0 & 0 & 0 & 0 & a_{76} & a_{77} \end{array}\right]\\ B(\Phi (t))= & {\left[\begin{array}{ccccccc} 1 & 0 & 0 & 0 & 0 & 0 & 0 \end{array}\right]}^{T} \end{array}$$

with the following output equation $y\left(t\right)=Q_{2}\left(t\right)/V_{g}$ measurable from the CGM sensor. The following terms have constant values

(15) $$\begin{array}{cc} a_{31}= & \frac{\eta k_{a}S_{IT}^{f}k_{a1}}{V_{i}k_{e}}\\ a_{41}= & \frac{-\eta k_{a}S_{IE}^{f}EGP_{0}}{V_{i}k_{e}}\\ a_{47}= & -a_{66}=a_{76}=-a_{77}=\frac{1}{t_{maxG}} \end{array}$$

and the nonlinearities can be embedded in the following terms

(16) $$\begin{array}{cc} a_{43}= & -Q_{1}\left(t\right)\\ a_{44}= & \frac{F_{01}^{c}\left(G\left(t\right)\right)}{Q_{1}\left(t\right)}-F_{R}\left(G\left(t\right)\right)\\ a_{45}= & \frac{EGP_{0}}{Q_{2}}\\ a_{52}= & \frac{\eta k_{a}S_{ID}^{f}}{V_{I}k_{e}}Q_{2}\left(t\right)\\ a_{53}= & Q_{2}\left(t\right) \end{array}$$

Furthermore, if measurements of Φ are available, and its admissible range of operation is known

(17) $${\underline{\Phi }}_{i}\leq \Phi_{i}\leq {\bar{\Phi }}_{i},\;i=1,\cdots ,l$$

where ${\underline{\Phi }}_{i}$ and ${\bar{\Phi }}_{i}$ are the lower and upper bounds of each element in Φ, then we can put the parameter vector Φ into polytopic form [44,45]

(18) $$\Phi \in Co\left\{\omega_{1},\omega_{2},\cdots ,\omega_{N}\right\}:=\left\{\sum_{i=1}^{N}\mu_{i}\omega_{i}:\;\mu_{i}\geq 0,\sum_{i=1}^{N}\mu_{i}=1\right\}$$

where $N=\left\{1,\cdots ,2^{n_{\Phi }}\right\}$. This transformation is known as the bounding box method, because Φ is the convex hull generated by vertex $\omega_{i}$. Hence, a polytopic representation of the system is obtained by the state space matrices defined at the different vertex of the convex hull

(19) $$\left[A\left(\Phi \right)\right]\in Co\left\{\left[A_{i}\right]:=\left[A_{i}\left(\omega_{i}\right)\right]\right\}$$

Using this approach, the system (12) can be represented as a weighting function of the system matrices at the polytope vertices

(20) $$\begin{array}{cc} \dot{x}\left(t\right)= & \sum_{i=1}^{2^{n_{\Phi }}}\left(\mu_{i}\left(\Phi \right)\right)\left(A_{i}\left(\Phi \right)x\left(t\right)+B_{i}\left(\Phi \right)u\left(t\right)\right)\\ y(t)= & \sum_{i=1}^{2^{n_{\Phi }}}\left(\mu_{i}\left(\Phi \right)\right)\left(C_{i}\left(\Phi \right)x\left(t\right)+D_{i}\left(\Phi \right)u\left(t\right)\right) \end{array}$$

where μ(i) are the membership functions. Basically, at any given time instant the state space system description is obtained by a linear interpolation of the system matrices at the polytope vertices. The weighting function is defined as in [46]

(21) $$\mu_{i}\left(\Phi \right)=\prod_{j=1}^{2^{n_{\Phi }}}\xi_{ij}\left(\eta_{0}^{j},\eta_{1}^{j}\right),\forall i=\left\{1,\cdots ,2^{n_{\Phi }}\right\}$$

with

(22) $$\xi_{ij}\left(\eta_{0}^{j},\eta_{1}^{j}\right)=\left\{\begin{array}{cc} \eta_{0}^{j}, & \mathrm{if}mod\left(n,2^{m}\right)\in \left\{1,\cdots ,2^{j-1}\right\}\;\\ \eta_{1}^{j}, & \mathrm{otherwise} \end{array}\right.$$

(23) $$\eta_{0}^{j}=\frac{{\bar{\Phi }}_{j}-\Phi \left(k\right)}{{\bar{\Phi }}_{j}-{\underline{\Phi }}_{j}}$$

(24) $$\eta_{1}^{j}=1-\eta_{0}^{j}$$

where $j=1,\cdots ,n_{\Phi }$ and each element of the vector Φ is known and varies in a known interval $\Phi_{j}\left(k\right)\in \left[{\underline{\Phi }}_{j},{\bar{\Phi }}_{j}\right]$. The scheduling variables for the reduced Hovorka model can be defined as

(25) $$\Phi \left(t\right)=\left[\begin{array}{cc} Q_{1}\left(t\right) & Q_{2}\left(t\right) \end{array}\right]$$

leading to four linear time invariant (LTI) systems that will conform the vertices of the polytope. Notice that this representation is not unique and other scheduling variables can be chosen depending on the model. The operational range for these variables is attached in Table 2.

The original continuous time system can be discretized using a number of discretization methods. In this work, we use the zero order hold approach with a sampling time of 5 min, which is a common measurement rate provided by CGM devices. Then, the continuous polytopic system (20) takes the following discrete-time representation

(26) $$\begin{array}{cc} x(k+1)= & \sum_{i=1}^{2^{n_{\Phi }}}\left(\mu_{i}\left(\Phi \right)\right)\left(A_{d,i}\left(\Phi \right)x\left(k\right)+B_{d,i}\left(\Phi \right)u\left(k\right)\right)\\ y(k)= & \sum_{i=1}^{2^{n_{\Phi }}}\left(\mu_{i}\left(\Phi \right)\right)\left(C_{d,i}\left(\Phi \right)x\left(k\right)+D_{d,i}\left(\Phi \right)u\left(k\right)\right) \end{array}$$

where $A_{d,i}\left(\Phi \right)\in R^{n_{x}\times n_{x}}$, $B_{d,i}\left(\Phi \right)\in R^{n_{x}\times n_{u}}$, $C_{d,i}\left(\Phi \right)\in R^{n_{y}\times n_{x}}$ and $D_{d,i}\left(\Phi \right)\in R^{n_{y}\times n_{u}}$ are the discretized state space matrices for the *i*-th vertex.

## 5. Residual Generation

The AP systems are characterized by having few sensors and actuators. Therefore, to monitor the system and patient state, observers are designed for the purpose of generating residuals. However, only little information is available for AP systems. In most cases, the only information available from sensors is the CGM glucose measurements. To tackle this limitation, the design of a robust state estimator for residual generation has been considered in this section.

### 5.1. Design Method

Residual generation is based on the state estimation using the polytopic LPV model (26) by means of a Kalman filter of the following form

(27) $$\begin{array}{cc} \hat{x}\left(k+1\right)= & \sum_{i=1}^{2^{n_{\Phi }}}\left(\mu_{i}\left(\Phi \right)\right)\left(A_{d,i}\left(\Phi \right)x\left(k\right)+B_{d,i}\left(\Phi \right)u\left(k\right)\right)+\\ \; & +L\left(\Phi \right)\left(y\left(k\right)-\hat{y}\left(k\right)\right) \end{array}$$

were $\hat{x}\in R^{n_{x}}$ are the estimated states and $L\left(\Phi \right)\in R^{n_{x}\times n_{y}}$ is the observer gain defined as

(28) $$L\left(\Phi \right)=\sum_{i=1}^{2^{n_{\Phi }}}\mu_{i}\left(\Phi \right)L_{i}$$

and $L_{i}$ are the observer gains for each one of the systems at the vertices of the polytope. Prior to the observer design, the observability property is checked. A total of $2^{n_{\Phi }}$ observability matrices are generated, for each one of the vertex polytopic representations

(29) $$O=\left[\begin{array}{c} C_{d,i}\left(\Phi \right)\\ C_{d,i}\left(\Phi \right)A_{d,i}\left(\Phi \right)\\ \vdots \\ C_{d,i}\left(\Phi \right)A_{d,i}{\left(\Phi \right)}^{n_{x}-1} \end{array}\right]$$

then, the system is observable if each observability matrix is of full rank, i.e., $rank\left(O\right)=n_{x}$.

The observer gain can be designed by solving the associated LQC dual problem [47]. Given the system description (26), tuning matrices $Q=Q^{T}=H^{T}H\geq 0$, $R=R^{T}>0$ and the performance bound γ. Then, the observer gains for the polytopic system are obtained by finding Υ and $W_{i}$ through the solution of the following LMI minimization problem

(30) $$\begin{array}{c} \min_{\gamma ,\Upsilon =\Upsilon^{T},W}\gamma \;\;\;\;\;\;\;\;\;\;\\ \mathrm{subject}\;\mathrm{to}\;\;\;\;\;\;\;\;\;\; \end{array}$$

(31) $$\left[\begin{array}{cc} \gamma I_{n} & I_{n}\\ I_{n} & \Upsilon \end{array}\right]>0$$

(32) $$\left[\begin{array}{cccc} -\Upsilon & \Upsilon A_{d,i}-W^{T}C_{d,i} & \Upsilon H^{T} & W^{T}\\ A_{d,i}^{T}\Upsilon -C_{d,i}^{T}W & -\Upsilon & 0 & 0\\ H\Upsilon & 0 & I_{n_{x}} & 0\\ W & 0 & 0 & -R^{-1} \end{array}\right]<0$$

(33) $$\left[\begin{array}{cc} -r\Upsilon & q\Upsilon +A_{d,i}^{T}\Upsilon -C_{d,i}^{T}W\\ q\Upsilon +\Upsilon A_{d,i}-WC_{d,i} & -r\Upsilon \end{array}\right]<0$$

where q=0 and r=1 are the center and radius of a unitary circle respectively and $I_{n_{x}}$ is the identity matrix of size $n_{x}$. Note that constraints (33) are not mandatory to solve the original LQC problem [47]. However, they guarantee the stability of the observer system.

In this paper, the process disturbances ($\omega \in R^{n_{x}}$) and measurement noise ($\upsilon \in R^{n_{y}}$) are unknown but assumed to be bounded and represented by zonotopes

(34) $$\begin{array}{c} W=\left\{\omega_{k}\in R^{n_{x}}:\left|\omega_{k}-c_{\omega }\right|\leq \bar{\omega },c_{\omega }\in R^{n_{x}},\bar{\omega }\in R^{n_{x}}\right\}=\langle c_{\omega },R_{\omega }\rangle \end{array}$$

(35) $$\begin{array}{c} V=\left\{\upsilon_{k}\in R^{n_{y}}:\left|\upsilon_{k}-c_{\upsilon }\right|\leq \bar{\upsilon },c_{\upsilon }\in R^{n_{y}},\bar{\upsilon }\in R^{n_{y}}\right\}=\langle c_{\upsilon },R_{\upsilon }\rangle \end{array}$$

where $c_{\omega }$ and $c_{\upsilon }$ are the centers of the process disturbances and measurement noise zonotopes, with the generator matrices being $R_{\omega }\in R^{n_{x}\times n_{x}}$ and $R_{\upsilon }\in R^{n_{y}\times n_{y}}$. Then, according to [48] the polytopic observer in (27) can be converted into a zonotopic state estimation observer by computing the center and the generator matrix of the state observer, i.e., $\hat{X}=\langle c_{x},R_{x}\rangle$, at every single step as follows

(36) $$\begin{array}{cc} c_{x}\left(k+1\right) & =c_{p}\left(k\right)+L\left(y\left(k\right)-\sum_{i=1}^{2^{n_{\Phi }}}\left(\mu_{i}\left(\Phi \right)C_{i}\right)c_{p}\left(k\right)\right) \end{array}$$

(37) $$\begin{array}{cc} R_{x}\left(k+1\right) & =\left[\left(I-L\sum_{i=1}^{2^{n_{\Phi }}}\left(\mu_{i}\left(\Phi \right)C_{i}\right)\right)R_{p}\left(k\right)\;\;-LE_{v}\right] \end{array}$$

with L being defined in Equation (28) and

(38) $$\begin{array}{cc} c_{p}\left(k+1\right) & =\sum_{i=1}^{2^{n_{\Phi }}}\left(\mu_{i}\left(\Phi \right)\right)\left(A_{i}\left(\Phi \right)x\left(k\right)+B_{i}u\left(k\right)\right) \end{array}$$

(39) $$\begin{array}{cc} R_{p}\left(k+1\right) & =\left[\sum_{i=1}^{2^{n_{\Phi }}}\left(\mu_{i}\left(\Phi \right)\right)\left(A_{i}\left(\Phi \right)\right)R_{x}\left(k\right)\;\;\;E_{\omega }\right] \end{array}$$

where $E_{\omega }$ and $E_{v}$ are the distribution matrices of the state disturbance and measurement noise vectors, respectively.

### 5.2. The Bank of Observers

The bank of interval zonotopic observers is designed to generate appropriate residual signals. The residual signals have to be sensitive to specific mode changes. Therefore, several observers based on variations of the reduced Hovorka model have been considered. Each of the observers tackle one specific mode of operation. A total of four different observers have been designed $\left(n_{o}=4\right)$:

1. *Meal observer*: Observer designed to track meal and postprandial periods for announced meals. It allows the system to detect faults in meal estimations and erroneous boluses. It includes the whole Hovorka model with two extended states related to CHO consumption. Its state space vector is defined by $x\left(k\right)={\left[S_{1}\;S_{2}\;x_{1}\;Q_{1}\;Q_{2}\;D_{1}\;D_{2}\right]}^{T}$. The CHO model time constant $t_{maxG}$ is set to 40 min as in [40];
2. *Resting and fasting observer*: Observer designed to track night and in-between disturbances periods. Its state space vector is defined by $x\left(k\right)={\left[S_{1}\;S_{2}\;x_{1}\;Q_{1}\;Q_{2}\right]}^{T}$. We assume that sleeping periods are characterized by having no disturbances affecting the system and/or the effect of previous disturbances is small. For this reason, the observer is designed considering only insulin inputs. Consistent estimations should be provided by this observer during periods similar to steady state periods, i.e., resting or fasting periods;
3. *Altered insulin sensitivity observer*: Observer designed to track periods with increased sensitivity, for example during aerobic exercise sessions. The model parameter η has been increased by a trial and error procedure to match simulated aerobic exercise sessions based on clinical results [32]. Its state space vector is defined by $x\left(k\right)={\left[S_{1}\;S_{2}\;x_{1}\;Q_{1}\;Q_{2}\right]}^{T}$;
4. *Rescue carbohydrates observer*: Observer designed to detect a specific patient-in-the-loop control action. The presented control approach uses CHO as a counter-regulatory action to prevent hypoglycemia. This observer takes the insulin and CHO controller actions as its inputs. The goal of the observer is to monitor the adherence of the patient to the rescue CHO. Its state space vector is defined by $x\left(k\right)={\left[S_{1}\;S_{2}\;x_{1}\;Q_{1}\;Q_{2}\;D_{1}\;D_{2}\right]}^{T}$. The CHO model time constant $t_{maxG}$ is set to 20 min as in [27].

Each of the designed observers return a full state space interval estimation ${\hat{x}}_{1},\;{\hat{x}}_{2},\ldots {\hat{x}}_{n_{o}}$. Then, using those estimations, the interval residuals are computed based on the state bounding zonotope $\hat{X}\left(k\right)$ with the following residual center $\left(c_{r}\right)$ and generator matrix $R_{r}$

(40) $$c_{r}\left(k\right)=y\left(k\right)-\sum_{i=1}^{2^{n_{\Phi }}}\left(\mu_{i}\left(\Phi \right)C_{i}\right)\hat{x}\left(k\right)$$

(41) $$R_{r}\left(k\right)=\left[-\sum_{i=1}^{2^{n_{\Phi }}}C_{i}R_{x}\left(k\right)-E_{v}\right]$$

Hence, the FD test is based on checking the following condition

(42) $$0\notin <c_{r}\left(k\right)\;R_{r}\left(k\right)>$$

A fault (or change of mode) is triggered when 0 is not included in the zonotope $<c_{r}\left(k\right)\;R_{r}\left(k\right)>$. To reduce the computational burden, condition (42) can be simplified to check whether or not 0 is included in a box enclosing the zonotope

(43) $$0\notin <c_{r}\left(k\right)\;b\left(R_{r}\left(k\right)\right)>$$

where $b\left(R_{r}\left(k\right)\right)=diag\left(\left|\right|R_{r}\left(k\right){\left|\right|}_{1}\right)$ and $\left|\right|\cdot {\left|\right|}_{1}$ is the element by element absolute value operator. Then, the zonotopic interval upper and lower bands used for FD in the FSM in Equation (3) are defined as

(44) $$\begin{array}{cc} t_{r_{i}}^{u}\left(k\right)= & c_{r}\left(k\right)+\left|\right|R_{r}\left(k\right){\left|\right|}_{1} \end{array}$$

(45) $$\begin{array}{cc} t_{r_{i}}^{l}\left(k\right)= & c_{r}\left(k\right)-\left|\right|R_{r}\left(k\right){\left|\right|}_{1} \end{array}$$

**Figure 4:**
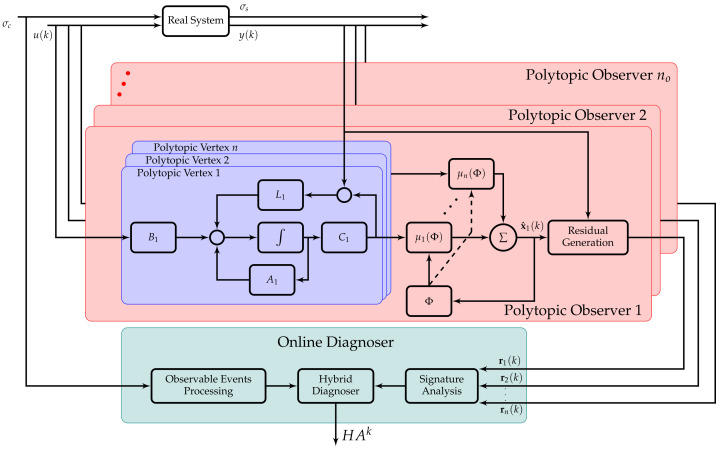
Conceptual diagram of the patient monitoring and FD system.

The overall method scheme of the fault diagnosis system is shown in Figure 4. The observer design problem is solved offline and the observer gains are obtained. For the online observer iterations, a continuous-discrete approach can be used. With this approach the a priori state estimation is obtained by integrating the original non-linear model, for example using the *ode45* function from Matlab. Then, the polytopic observer gain is found by interpolation using (28). Residuals are then generated for each of the observers and fed to the signature analysis module.

## 6. Results

### 6.1. In Silico Benchmarks

To test the robustness and precision of the monitoring system, two in silico benchmarks have been developed. Each of the benchmarks were designed to check the proper behavior of the system and its ability to detect different patient-in-the-loop modes and faults. All simulations have been executed with Matlab R2019a using an AMD Ryzen 3800× 3.9 GHz processor with 32 GB of RAM.

The FDA accepted UVa/Padova T1D Simulator (v3.2) [49] was used to evaluate the strategy. All of the benchmarks share some scenario settings, which include a virtual cohort of 10 adult patients. Scenarios last 4 days and 3 mixed meals of 60, 80, and 70 grams are included each day at 8:00, 13:00, and 21:00, respectively. Intra-subject variability in insulin absorption was included by assuming a ±30% in parameters $\left(k_{d},\;k_{a1},\;k_{a2}\right)$, and variability in insulin sensitivity was modeled by a sinusoidal pattern in parameters $\left(V_{mx},k_{p3}\right)$ [50]. The first scenario is designed to evaluate performance when only meal disturbances appear. The second scenario includes one session of aerobic exercise of heavy intensity (60% VO_2max_) during the second scenario day at 18:00 for a total duration of 50 min. Exercise was included into the simulation by using a previous developed model, which was fit to clinical data, that increases insulin sensitivity [32,51].

### 6.2. Patient Mode Detection

Scenarios 1 and 2 were used to perform four complete simulations, which consider different patient-in-the-loop faults. The simulations have the following characteristics: (1) scenario 1 without faults, (2) scenario 2 without faults, (3) scenario 1 with patient misestimated meals, and (4) scenario 2 with misestimated meals and missed rescue CHO. In total, 480 meals were consumed and 20 exercise sessions were performed across all simulations. Faults were introduced in a total of 160 meals, with over- and underestimations of ±60%, and feed-forward rescue CHO were also simulated with patient-in-the-loop faults in 10 occasions.

Meals were correctly detected in 461 occasions out of the total 480 meals across all simulations, resulting in a high sensitivity of 96.0%. Transitions towards the meal mode $q_{1}$ were triggered by the announcements and validated by the meal residual observer, resulting in a detection time from the start of the event of 13 min, while the average time in meal and postprandial period was 158 min. All exercise sessions were correctly detected without false positives/negatives. The effect of exercise is known to last during the following hours after an exercise session, this is also reflected in the time the system remained on mode $q_{2}$ of 328 min. Rest periods were considered as the periods in-between meals, without periods where exercise happened, or during night. No detection time for these periods is provided since there is no possible definition of when these events started, average time in this period was 328 min.

Transitions to faulty modes only happened in simulations 3 and 4. Faults in the consumption of rescue CHO, leading to transitions towards mode $q_{4}$, were recorded from simulation 4. A total of 10 missed rescue CHO consumption events were considered at the instant of exercise announcement at 17:40 of simulation day 2. The HA correctly detected 8 of these faulty events and was also able to transition afterwards to the $q_{2}$ mode. Two of the faulty events were not detected and the HA automaton directly transitioned to the altered insulin sensitivity mode $q_{2}$, indicating a exercise. Misestimated meals were correctly detected in 73 occasions with a sensitivity of 45.6%. No transitions to state $q_{5}$ happened.

**Figure 5:**
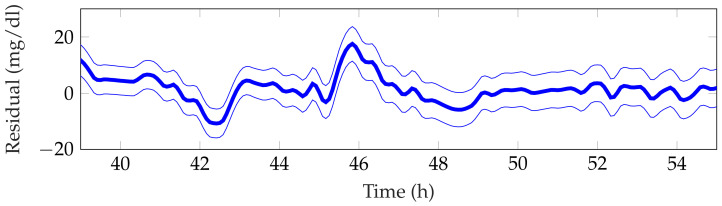
Representative fast and resting observer residual for the period 39–55 h from patient 10.

**Figure 6:**
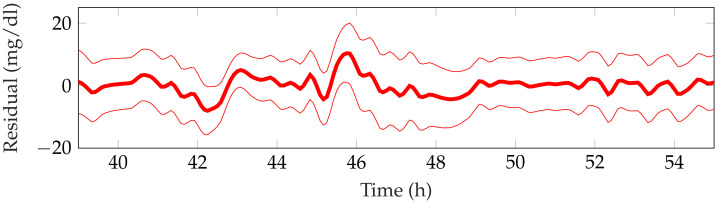
Representative meal observer residual for the period 39–55 h from patient 10.

**Figure 7:**
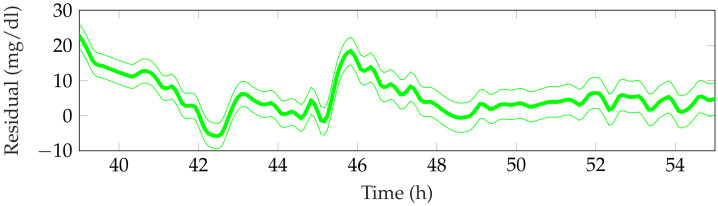
Representative altered insulin sensitivity observer residual for the period 39–55 h from patient 10.

**Figure 8:**
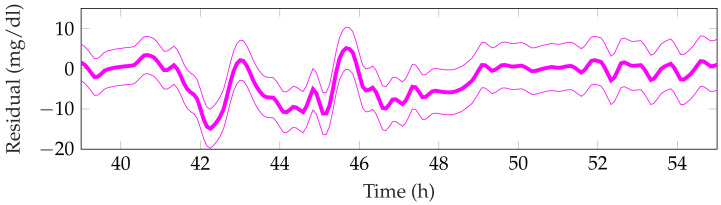
Representative rescue carbohydrates observer residual for the period 39–55 h from patient 10.

**Figure 9:**
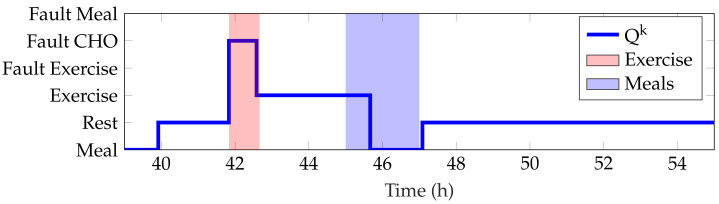
Representative detection of faulty rescue CHO by the automaton state $Q^{k}$ for patient 10. Data shown correspond to the simulation period from 39 to 55 h. The meal period comprises the 2 h following the meal and the exercise period includes the active exercise and the time in advance of the exercise announcement.

Table 3 shows the detection performance of the proposed approach for the aggregated four simulations. Figure 5, Figure 6, Figure 7, Figure 8 and Figure 9 showcase a simulation portion (from 39 to 55 simulation hours) for the adult patient 10. Exercise starts at time instant 42 h and feed-forward CHO are suggested at 39 h and 40 min. In this, case the patient does not consume the recommended amount of CHO and the system transitions to a faulty CHO state. Notice, that the system does not know if the patient has consumed or not the suggested rescue CHO. Afterwards, the system transitions to the exercise state and remains in that state for the following hours. This is an expected behavior of the system due to the modified insulin sensitivity that last several hours after the end of exercise. Then, at time instant 45 h the patient consumes a meal but the HA state remains at exercise. That is a normal behavior due to the intertwined disturbance effects on the residuals.

### 6.3. Example of Controller Reconfiguration

This section provides insights regarding the benefits of using the proposed HA model for the detection of patient-in-the-loop faults and controller reconfiguration. Particularly, we focus on the case of announced exercise. When people using an AP announce exercise, the feed-forward control can be applied in advance to mitigate the risk of exercise induced hypoglycemia. The AP used in this work has an embedded feed-forward controller for exercise [32]. This block may degrade performance if triggered by an exercise announcement from the patient, but no physical activity is performed afterwards. To test the benefits of controller reconfiguration, we use scenario 1, which does not include exercise, and assume exercise at 16:00 during the second simulation day.

The actions triggered by the feed-forward block are: (1) suggestion of feed-forward CHO, (2) basal insulin reduction, (3) reduction of the next insulin bolus, and (4) decrease of the insulin controller aggressivity (lower controller gains for the next 6 h after the ending of the exercise session). Controller reconfiguration happens if there is a mode detection change to mode $q_{5}$ in a timely manner and the CGM is higher than 150 mg/dL. We assume exercise is announced 20 min prior to the start [32] and we let the system monitor the patient until 20 min after the supposed start of exercise. If the $HA^{k}$ is in a normal operational mode and there is no mode change detection to $q_{2}$ or a mode transition to $q_{5}$ happens in that period of time, then the controller resumes its original tuning and assumes that the patient is not exercising even though there was an announcement.

**Figure 10:**
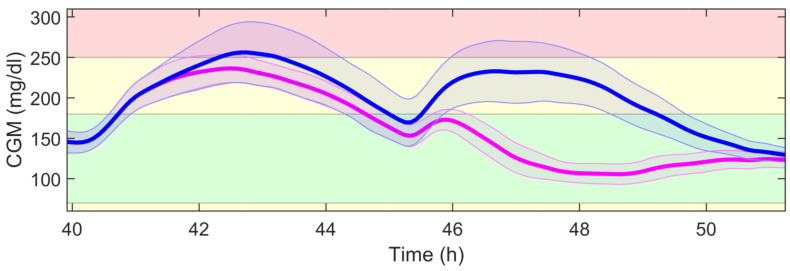
Population CGM trajectory (MEAN ± STD) when an exercise announcement patient-in-the-loop fault affects the system. The blue curve represents the controller without reconfiguration and the magenta curve represents the controller with reconfiguration. The exercise announcement occurs at time instant 39 h and 40 min.

**Figure 11:**
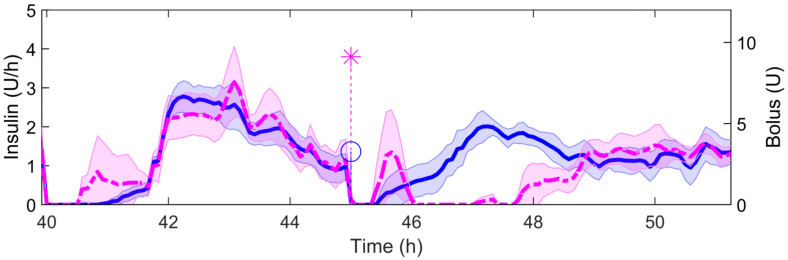
Population insulin trajectory (MEAN ± STD) when an exercise announcement patient-in-the-loop fault affects the system. The blue curve represents the controller without reconfiguration and the magenta curve represents the controller with reconfiguration. The circular and star points at the 45th simulation hour represent the mean boluses taken in each simulation.

Table 4 shows the glycemic results when comparing the strategies with (AP+HA column) and without (AP column) controller reconfiguration when faults in exercise announcement happen. Figure 10 and Figure 11 show the CGM trajectories and insulin infusions for each case. The patient announces exercise at time instant 39 h and 40 min (day 2, 15:40). The patient is supposed to start exercising at 40 h (day 2, 16:00) and have dinner at 45 h (day 2, 21:00). Results show that reconfiguration allows for early insulin infusion to start the following meal with lower BG and allows for better and tighter BG control during the postprandial period following an exercise session.

Controller reconfiguration allowed for a tighter and better BG control during and after the false exercise session and also for the next postprandial period. Overall, time in range 70–180 mg/dL significantly increased from 38.3 (34.3, 47.5)% to 62.0 (58.4, 71.5)% and the mean CGM was also reduced from 211.7 (172.4, 231.9) mg/dL to 154.9 (121.0, 206.3) mg/dL. The risk of hyperglycemia was also minimized as reflected by the time ranges above 180 mg/dL, and no risk of hypoglycemia was observed. Insulin infusion was resumed earlier upon mode detection as shown in Figure 11, allowing lower BG at the start of the second day dinner. Postprandial control was specially improved in the reconfigured controller, mainly due to the restored insulin bolus previously mitigated by the exercise feedforward actions.

## 7. Discussion

The patient-in-the-loop concept is fundamental for any diabetes treatment. Patients play essential roles within the treatment, from being the plant to control to actuate as an operator. This is the case for both OL and CL insulin therapies, and significantly more important in OL strategies where patients have more responsibility. Errors introduced by patients may compromise the performance and stability of treatments and patients may put themselves at risk. To minimize patient-in-the-loop faults, tools that monitor and detect those behaviors are needed. Detection of faults may allow CL controllers to take additional corrective actions to minimize the impact of the fault on the system performance. However, not all patient-in-the-loop faults might be easy to respond to. In any situation, monitoring systems will allow the collection of patient data that could be used to individualize the tuning of controllers or identify when faults are most likely to happen. Additionally, the detection of patient-in-the-loop faults with minimal impact during CL operation can be hard. CL systems are designed to reduce variability of the controlled variable and for disturbance rejection. Therefore, faults that have a small impact on the system and on BG concentration might be counteracted by the CL system itself and not detected as a fault. This is the case of misestimated meals in hybrid AP settings. In the case of OL therapies, this issue should be of less importance, allowing for more accurate detection.

To tackle this issue, we proposed a methodology based on a HA model. Even though the results we obtained were satisfactory and promising, our study suffered from several limitations. The first one is related to the data used to classify patient modes and faults. We used a simulation environment to stress the system and check its robustness and performance in several proof-of-concept examples. Certain limitations exist when trying to replicate real patient-in-the-loop faults in a simulation environment. BG trajectories may significantly vary depending on what the patient is performing, which may fall under normal operation. For example, postprandial periods where the patient is mostly in a sedentary state may differ from periods where the patient engages in slow walks. In any situation, these should not be detected as different patient states since there is no abnormality in the system. Real free-living condition data is still needed to assess the performance and detection capabilities of the proposed approach.

In this approach, we used a bank of interval zonotopic Kalman filters for the purpose of generating residuals. There are limitations to this approach due to the fact of using an approximate model and limited measurements. Particularly, the altered sensitivity observer has been designed to detect decreases in BG caused by aerobic exercise. Other types of exercise may have different effects on insulin sensitivity, requiring additional observers. Other factors such as illness, stress, menstruation cycle, sleep apnea, or some medications can also lead to important physiological changes, including changes in insulin sensitivity [52,53]. Therefore, the system is assumed to be working under normal operation for the detection of aerobic exercise. The major limitation of using a bank of observers is the need to build many different observers for the detection of the multitude of factors encountered in free living conditions. We used a reduced glucoregulatory model to relax the problem of observability of linear systems when only one measurement is available, but many states have to be estimated. Even though we only used the observers as a mean to generate residuals, the information from the observers could also be used to estimate other physiological parameters, such as the rates of glucose absorption.

We used the basic AP configuration, which only includes a CGM and an insulin pump. This was decided because even though there are studies using additional signals (such as using heart rate monitors, energy expenditure information and galvanic skin responses) these additional signals mean that the user (the patient) will have to wear additional sensors. Thus, although this could be of benefit for better detection performance, it also means that there is an increased risk of faults because more devices are used. Ultimately, the CGM and an insulin pump are always required devices for any AP operation. The proposed approach has two goals: (1) provide information for the AP in real time, and (2) provide information for long term decision making. By classifying patient errors, we believe that the system can be fine-tuned to maximize performance and minimize the risk of hypoglycemia due to patient actions. The developed approach could also work with different AP configurations, for example, AP systems with additional control signals such as glucagon [54]. This holds true because the developed approach is model-based, and by adding extra control actions the real model (the patient) retains the same dynamics. However, notice that this may require a re-tuning of the approach since not all possible modes have been considered, for example, faults regarding glucagon. The system performance may be compromised if used with other treatment options such as cadaveric islet or stem-cell derived islet transplantation. This also holds true when using the proposed approach for very specific sub-cohorts of people with T1D. All approaches that may modify the open-loop dynamics of the patient will require a model check for the proposed system to work.

Automated insulin delivery systems are still under research and improvement, with few systems such as the Medtronic 670 G and newer 780 G available on the market [55]. The most common AP uses the so-called subcutaneous route to sense glucose and deliver insulin. Non-physiological routes inevitably create non-physiological delays that are nonexistent in the healthy endogenous beta cells when sensing glucose and secreting insulin [56]. The major limiting factor of the subcutaneous route is related to the absorption of fast or ultra-fast insulin analogues. Most of these insulin formulations start actuating 10–15 min after their injection with a maximum peak of action between 1 and 2 h [57]. One of the consequences of this is the fact that most AP systems are rather conservative in insulin delivery to avoid overdoses of insulin, which may lead to hypoglycemia. As new insulins come to the market with faster profiles, the AP systems should be able to better control blood glucose due to a lowered delay of the control action effect. For example, newer formulations of insulin Aspart such as AT247, IAsp and FIAsp still have an approximate onset of exposure of 5 min [58,59,60]. On the other hand, CGM sensors have gradually improved their accuracy and some models can already be used as non-adjunctive, such as de Dexcom G6 [61]. Delays in blood glucose sensing by CGMs have been acknowledged for a long time [62]. Newer models already reported MARD < 10%, however, delays between plasma insulin and measured CGM glucose concentrations are still in the range of 5–10 min [61]. CGM technology provides close to continuous flux of glucose readings, generally between 1 and 5 min. Although this measurement sampling rate is much slower compared to how fast the healthy endogenous beta cells sense glucose, it is fast enough to control blood glucose since the dynamics of the overall system are much slower.

Current AP systems will certainly improve their performance if the delays associated with the subcutaneous route are reduced [56]. Especially, in situations where fast disturbances affect the system, for example for postprandial control [63]. Recent clinical trial outcomes of AP systems suggest that night periods are better controlled compared to traditional therapies [8], while day periods remain a challenge. This is caused by the absence of disturbances, mainly meals, during nights. Therefore, controlling postprandial periods is still difficult for AP systems even with the newest improved algorithms. Improving the absorption of insulin analogues will most likely allow for better control of postprandial periods than improving the already tested algorithms. The proposed approach should be able to accommodate improvements for newer insulin formulations and CGM systems since it is model-based. Theoretically, these improvements are equivalent to changing the time constants of the insulin and glucose sub-systems change. Then, the proposed approach would require including these new time constants in the models used. Clearly, the model change will have to be validated and the system performance assessed again.

Detection performance should be analyzed in depth before performing controller reconfiguration. While some patient-in-the-loop faults might be easier to detect with high accuracy, others might show higher rate of FP and FN. The results presented in this paper showed that detecting faults in CHO control actions can be accurately performed, while detecting errors in meal estimation is harder. The detection of meal misestimations is especially critical for the case of FP events, where controller reconfiguration could be triggered erroneously. This is something that must be taken into consideration when designing the FTC strategy. Detecting faults of people with type 1 diabetes is a complex task. Unfortunately, to discriminate different faults, the use of different observers is required. Nevertheless, the HA model is not restricted to work with observers. Residuals can be generated in a multitude of ways. In this work we used a bank of observers for the generation of residuals. However, any other methodology for the residual generation can be used and incorporated into the HA model.

## 8. Conclusions

A methodology to monitor patient-in-the-loop modes and faults has been presented. The system is built around a HA model that replicate patient’s real life operation modes. Transitions between different modes allow for the identification of key operational modes and faults. The diagnoser is responsible for mode recognition by using a group of indicators generated from a set of residuals for every mode. A bank of interval zonotopic Kalman filters was constructed for the residual generation, allowing the system to have confidence bounds on the state estimation and residual generation.

Several proof of concept simulated benchmarks were done using challenging scenarios. The results suggest that mode and patient-in-the-loop faults can be detected in real time. Next, the information resulting from this study could be used as a tool to reconfigure CL controllers, monitor the system continuously and classify patient-in-the-loop behaviors. The exploration of these areas will be part of future research. Moreover, the adaptation of the LPV Hovorka reduced model to represent glycemic dynamics will be investigated.

## Figures and Tables

**Table 1:** Model parameters.

Parameter	Value	Description
*B* (kg)	Individualized	Weight
$k_{12}\;\left(\min^{-1}\right)$	0.066	Transfer rate
$k_{a1}\;\left(\min^{-1}\right)$	0.006	Deactivation rate
$k_{a2}\;\left(\min^{-1}\right)$	0.06	Deactivation rate
$k_{a3}\;\left(\min^{-1}\right)$	0.03	Deactivation rate
$k_{e}\;\left(\min^{-1}\right)$	0.138	Time constant of insulin elimination
$V_{i}\;\left(L\right)$	0.12 BW	Insulin distribution volume
$V_{g}\;\left(L\right)$	0.16 BW	Glucose distribution volume
$S_{IT}^{f}\;\left(L\;\min^{-1}\;{\mathrm{mU}}^{-1}\right)$	$51.2\times 10^{-4}$	Insulin sensitivity on transport
$S_{ID}^{f}\;\left(L\;\min^{-1}\;{\mathrm{mU}}^{-1}\right)$	$8.2\times 10^{-4}$	Insulin sensitivity of disposal
$S_{IE}^{f}\;\left(L\;{\mathrm{mU}}^{-1}\right)$	$520\times 10^{-4}$	Insulin sensitivity of $EGP_{0}$
$EGP_{0}\;\left(\mathrm{mmol}\;{\mathrm{kg}}^{-1}\min^{-1}\right)$	0.0161	Endogenous glucose production at zero insulin
$F_{01}\;\left(\mathrm{mmol}\;{\mathrm{kg}}^{-1}\;\min^{-1}\right)$	0.0097	Non-insulin-dependant glucose flux
$t_{maxI}\;\left(\min^{-1}\right)$	55	Time constant of insulin absorption

**Table 2:** Limits for the system scheduling variables.

Variable	Minimum	Maximum
$Q_{1}$ (mmol)	10	400
$Q_{2}$ (mmol)	10	400

**Table 3:** Population performance metrics of patients mode transition by the HA.

Mode	TP	FP	FN	Sensitivity (%)	Mean Transition Time (min)	Mean Activated Time (min)
$q_{1}$	461	1	19	96.0	13	158
$q_{2}$	20	0	0	100.0	55	288
$q_{3}$	476	0	33	93.5	-	328
$q_{4}$	8	0	2	80.0	17	83
$q_{6}$	73	20	87	45.6	28	22

**Table 4:** Glycemic performance when subjects introduce faults in the exercise announcement.

Performance Indicator	AP	AP+HA
Mean CGM (mg/dL)	211.7(172.4,231.9)	$154.9\;{\left(121.0,206.3\right)}^{*}$
Median CGM (mg/dL)	199.8(164.4,221.1)	$153.7\;{\left(117.5,200.6\right)}^{*}$
Maximum CGM (mg/dL)	361.3(258.1,410.3)	$200.9\;{\left(174.0,265.3\right)}^{*}$
Minimum CGM (mg/dL)	142.9(125.9,162.1)	$108.2\;{\left(87.3,157.9\right)}^{*}$
% of time CGM		
>250 mg/dL	8.0(0.0,18.3)	0.0(0.0,16.8)
>180 mg/dL	51.1(35.8,52.6)	30.7(20.4,37.2)
70–180 mg/dL	38.3(34.3,47.5)	$62.0\;{\left(58.4,71.5\right)}^{*}$
<70 mg/dL	0.0(0.0,0.0)	0.0(0.0,0.0)
<54 mg/dL	0.0(0.0,0.0)	0.0(0.0,0.0)

The results are median (interquartile range) during the time period between the 40th and 51th simulation hours. * *p* value <0.01 (Wilcoxon signed rank test). AP refers to the system without controller reconfiguration and AP+HA refers to the system with controller reconfiguration.

## Abbreviations

The following abbreviations are used in this manuscript:

T1D	Type 1 Diabetes
BG	Blood Glucose
AP	Artificial Pancreas
CL	Closed-Loop
CGM	Continuous Glucose Monitoring
FTC	Fault Tolerant Control
FD	Fault Detection
FI	Fault Identification
CHO	Carbohydrates
LPV	Linear Parameter Varying
LQC	Linear Quadratic Control
LMI	Linear Matrix Inequalities
IO	Interval Observers
HA	Hybrid Automaton
IFB	Insulin Feedback
PD	Proportional-Derivative
IOB	Insulin-On-Board
SMRC	Sliding Mode Reference Conditioning
COB	Carbohydrates-On-Board
FDA	Food and Drug Administration
